# Periodic Endoscopies Might Not Increase the Detection of Early Gastric Cancer in a Young Population

**DOI:** 10.1371/journal.pone.0159759

**Published:** 2016-07-22

**Authors:** Chan Hyuk Park, Eun Hye Kim, Hyunsoo Chung, Jun Chul Park, Sung Kwan Shin, Yong Chan Lee, Ji Yeong An, Hyoung-Il Kim, Jae-Ho Cheong, Woo Jin Hyung, Sung Hoon Noh, Choong Bae Kim, Sang Kil Lee

**Affiliations:** 1 Department of Internal Medicine, Hanyang University Guri Hospital, Hanyang University College of Medicine, Guri, Korea; 2 Division of Gastroenterology, Department of Internal Medicine, Severance Hospital, Institute of Gastroenterology, Yonsei University College of Medicine, Seoul, Korea; 3 Department of Surgery, Severance Hospital, Yonsei University College of Medicine, Seoul, Korea; Baylor College of Medicine, UNITED STATES

## Abstract

**Background:**

Screening endoscopies in individuals 40 years or older in regions where gastric cancer is prevalent increase the diagnosis of gastric cancer at an early stage. However, the benefits of screening endoscopies in a young population (<40 years) have not been evaluated.

**Methods:**

We reviewed data from patients less than 40 years old who underwent endoscopic submucosal dissection or surgery for initial-onset gastric cancer. We also administered a questionnaire to gather information concerning periodic endoscopic inspections and the period from the penultimate endoscopy to diagnosis.

**Results:**

Of the 564 patients in this study, 101 (17.9%) patients underwent screening endoscopy within 24 months of their gastric cancer diagnosis. Lesion size was significantly smaller in the ≤24 months group than in the >24 month group (23.8 mm [standard deviation, 22.2 mm] vs. 30.5 mm [standard deviation, 23.1 mm], *P* = 0.008). However, the proportion of patients with early gastric cancer did not differ between the two groups (≤24 months vs. >24 months group; 67.6% vs. 65.7%, *P* = 0.712). On multivariable analysis, periodic endoscopies did not influence the early diagnosis of gastric cancer (with >24 months as the reference group: ≤24 months, odds ratio = 0.939, 95% confidence interval = 0.583–1.513).

**Conclusion:**

Although periodic endoscopies aided in the detection of gastric cancer when lesions were smaller in size, they seemed not to increase the proportion of patients with early gastric cancer in young patients diagnosed with resectable gastric cancer.

## Introduction

Gastric cancer is one of the major causes of cancer-related death worldwide, with almost 990,000 cases detected annually [1]. The prognosis of patients with gastric cancer depends on tumor stage [2–4]. In Korea and Japan, where gastric cancer is prevalent, a mass screening program that employs upper endoscopy and gastrofluoroscopy has been introduced to detect gastric cancer while it is still at an early stage [5,6]. The National Cancer Screening Program in Korea recommends biennial upper endoscopies or gastrofluoroscopies for individuals 40 years or older [6]. Our previous study revealed that biennial endoscopies increased the diagnosis of gastric neoplasms, including gastric cancer and adenoma, at an early stage in individuals 40 years or older [7].

Although gastric cancer most frequently develops after the age of 40, it can also occur in younger individuals (<40 years) [8–10]. A recently published study in Japan showed that the survival rate of young patients with gastric cancer was similar to that of middle-aged patients with gastric cancer [11]. Moreover, it was shown that the disease-free and overall survival of young patients with gastric cancer depended on cancer stage at diagnosis, as is the case with middle-aged patients with gastric cancer. Therefore, diagnosis of gastric cancer at an earlier stage is important for improving patient survival even though patients can undergo curative surgery.

Concerns about gastric cancer may lead to voluntary cancer screening in young people as well as in the elderly. In fact, 26% of the patients who underwent endoscopic screening at a health care center in Korea were young individuals less than 40 years of age [8,12], despite the lack of a recommendation for mass screening in this population by the National Cancer Screening Program in Korea [6]. However, gastric cancer in young patients displays different clinicopathologic features and molecular characteristics compared to gastric cancer in elderly patients; therefore, we cannot assume that periodic endoscopy in a young population will be beneficial for detecting gastric cancer at an earlier stage [10,11,13–18]. Undifferentiated gastric cancer is more common in young patients than in elderly patients [10]. The rapid progression of gastric cancer has also been suggested to be the reason for the poor prognosis of young patients [19]. If gastric cancer progresses more rapidly in younger patients, endoscopic screening may not be beneficial for early diagnosis.

To determine whether periodic endoscopy can aid in the detection of resectable gastric cancer at an earlier stage, we evaluated the proportion of young patients diagnosed with early gastric cancer (EGC) who underwent curative treatment according to their endoscopic examination history.

## Methods

We retrospectively analyzed patient demographics and clinical data. This study included patients less than 40 years old who underwent endoscopic submucosal dissection (ESD) or surgery for initial-onset gastric cancer at Severance Hospital in Seoul, Korea between January 2008 and April 2014.

Patients were asked a series of questions over the phone regarding their gastrointestinal symptoms at the time of diagnosis and whether they had undergone periodic endoscopies before being diagnosed. The three questions asked were (a) Did you have gastrointestinal symptoms, such as abdominal pain, discomfort, soreness, and dyspepsia, shortly before the gastric cancer diagnosis through the endoscopy? (b) Did you undergo an esophagogastroduodenoscopy before you were diagnosed with gastric cancer? and (c) If you underwent an esophagogastroduodenoscopy prior to diagnosis, how much time elapsed between the penultimate endoscopy and the diagnosis?

Among the patients initially included in the study, those who received preoperative chemotherapy or radiotherapy were excluded. Patients who could not be contacted over the telephone or who were unable to recall their endoscopy history were also excluded. Additionally, patients who underwent a penultimate endoscopy within 6 months of their gastric cancer diagnosis were excluded because it was possible that they underwent the endoscopy because of a misdiagnosis of symptomatic gastric cancer or an uncertain diagnosis of gastric cancer [7,20]. Because the National Cancer Screening Program in Korea, where gastric cancer is prevalent, recommends biennial screening for gastric cancer [6], enrolled patients were classified according to the endoscopy interval as follows: (1) ≤24 months and (2) no endoscopy within 24 months.

Patient records/information were anonymized and de-identified prior to analysis. Verbal informed consent was obtained from each participant before the telephone survey. The Institutional Review Board of Severance Hospital approved this study and the consent procedure.

### Treatment method

The standard treatment modality for gastric cancer without evidence of distant metastasis is radical gastrectomy with lymph node dissection. However, EGCs that met the following categories clinically were treated with ESD: tumors clinically diagnosed as T1a and (a) differentiated-type, ulcer (-), but >2 cm in diameter, (b) differentiated-type, ulcer (+), and ≤3 cm in diameter, or (c) undifferentiated-type, ulcer (-), and ≤2 cm in diameter [21]. Whether the lesion that had been treated with ESD met the ESD indication criteria was assessed by pathologic examination after ESD based on the expanded indication criteria, as proposed by Gotoda *et al*., as follows: (a) differentiated intramucosal adenocarcinoma smaller than 3 cm in diameter without lymphovascular invasion, irrespective of ulcer findings; (b) differentiated intramucosal adenocarcinoma without lymphovascular invasion and negative for ulceration, irrespective of tumor size; (c) undifferentiated intramucosal cancer smaller than 2 cm without lymphovascular invasion and ulcer findings; and (d) differentiated adenocarcinoma smaller than 3 cm with minimal submucosal invasion (<500 μm) and without lymphovascular invasion. Therefore, some lesions initially treated with ESD were later discovered to be beyond ESD indications. These patients subsequently underwent surgery as treatment for gastric cancer.

### Gross and histopathologic evaluation

Tumor location was endoscopically evaluated and categorized using the Japanese Gastric Cancer Association Classification criteria [22]. Tumor size, invasion depth, the presence of an ulcer, lymphatic and vascular involvement, and lymph node metastasis were histopathologically assessed. Pathological stages were determined according to the 7^th^ edition of the American Joint Committee on Cancer/Union Internationale Contre le Cancer tumor-node-metastasis staging system [23].

### Statistical analysis

Descriptive statistics of continuous variables are presented as the mean ± standard deviation. Statistical tests to compare results included *t*-tests, chi-square tests, and Fisher’s exact tests. Logistic regression analysis was performed to adjust for possible confounding variables that were discernible before esophagogastroduodenoscopy, including age, sex, first-degree relatives with gastric cancer, and gastrointestinal symptoms. In addition, we performed a retrospective power calculation for the primary endpoint (the proportion of EGC), because all relevant data other than a questionnaire were collected retrospectively. *P* < 0.05 indicated a significant difference between groups. All statistical analyses were performed using SPSS for Windows (version 18.0; SPSS Inc., Chicago, IL, USA).

## Results

**Fig 1:**
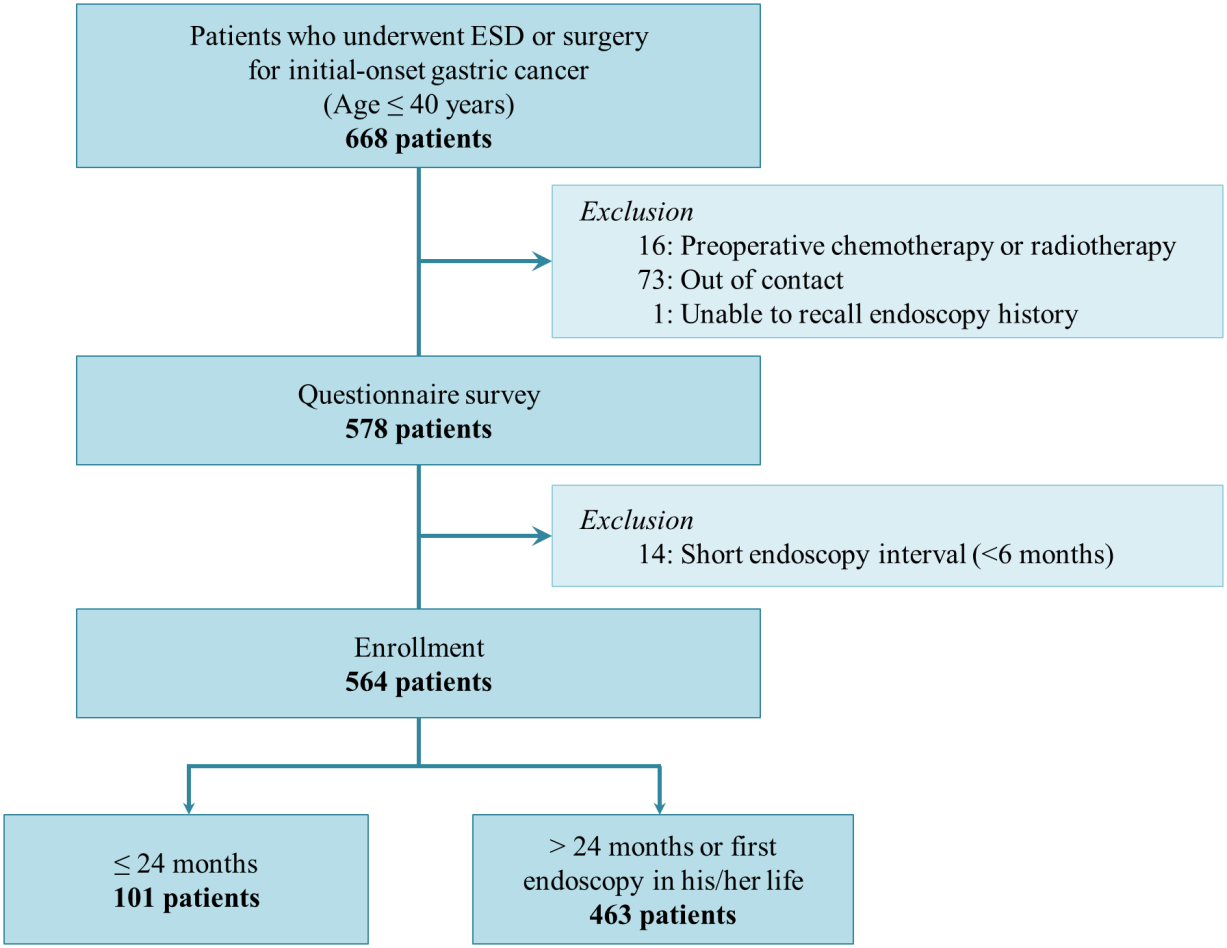
Flow diagram of the study design.

A flow diagram of the study design is shown in Fig 1. Among the 668 patients less than 40 years old who underwent ESD or surgery for initial-onset gastric cancer, 16 were excluded because they received preoperative chemotherapy or radiotherapy. Another 74 patients were excluded because they could not be contacted over the telephone or were unable to recall their endoscopy history. Additionally, 14 patients who underwent a penultimate endoscopy within 6 months of their diagnosis of gastric cancer were excluded. After these exclusions, a total of 564 patients were analyzed in this study.

### Baseline patient and lesion characteristics

Table 1 shows the baseline patient and lesion characteristics. The mean patient age was 36 years, and the proportion of male patients was 46.6%. The proportion of patients with first-degree relatives with gastric cancer was 8.7%. For the endoscopy interval, there were 101 (17.9%) patients with intervals of ≤24 months between endoscopy examinations, while 463 (82.1%) patients had not undergone an endoscopy within 2 years.

**Table 1:** Baseline patient and lesion characteristics.

Variable	Value
Patient, n	564
Lesion, n	569
Age, yr, mean ± SD	36.3 ± 3.8
Sex, n (%)	
Male	263 (46.6)
Female	301 (53.4)
First-degree relatives with gastric cancer, n (%)	49 (8.7)
Gastrointestinal symptom,^a^ n (%)	
Absent	247 (43.8)
Present	317 (56.2)
Interval between endoscopic examinations, months, n (%)	
≤ 24	101 (17.9)
> 24^b^	463 (82.1)
Location, n (%)	
Upper third	67 (11.8)
Middle third	271 (47.6)
Lower third	231 (40.6)
Lesion size, cm, n (%)	
≤ 2	257 (45.2)
2–5	238 (41.8)
> 5	74 (13.0)
Differentiation, n (%)	
Differentiated cancer	91 (16.0)
Undifferentiated cancer	478 (84.0)
Disease status, n (%)	
EGC	376 (66.1)
AGC	193 (33.9)
Stage, n (%)	
I	388 (68.2)
II	94 (16.5)
III	87 (15.3)
Possibility for ESD, n (%)	
Within indication	155 (27.2)
Beyond indication	414 (72.8)
Treatment modality, n (%)	
ESD	31 (5.4)
Surgery	538 (94.6)

^a^ Gastrointestinal symptoms included abdominal pain, discomfort, soreness, and dyspepsia.

^b^ This category included patients diagnosed with gastric cancer by the first endoscopy performed.

EGC, early gastric cancer; AGC, advanced gastric cancer; ESD, endoscopic submucosal dissection; SD, standard deviation.

Most of the lesions were located in the middle third of the stomach (47.6%) and represented undifferentiated cancer (84.0%). At presentation, 66% of the lesions were EGC. Although 27.2% of the lesions fulfilled the expanded indication criteria for ESD, only 5.4% were treated with ESD.

### Impact of periodic endoscopy

**Fig 2:**
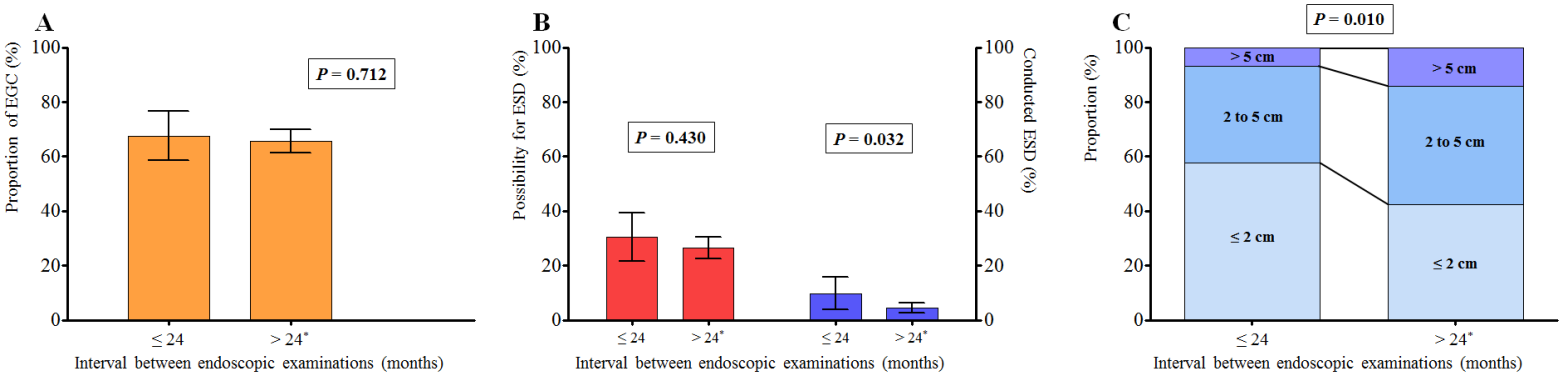
(A) The proportion of patients with early gastric cancer according to the interval between endoscopic examinations. (B) The proportion of lesions that fulfilled the expanded indications for endoscopic submucosal dissection (red) and lesions treated with endoscopic submucosal dissection (blue) according to the interval between endoscopic examinations. Lesion size according to the interval between endoscopic examinations. Sky blue, ≤2 cm; deep sky blue, 2–5 cm; slate blue, >5 cm. *This category included patients diagnosed with gastric cancer by the first endoscopy performed. EGC, early gastric cancer; ESD, endoscopic submucosal dissection.

The main outcome of the study, which was the proportion of patients with EGC according to the interval between endoscopic examinations, is shown in Fig 2A. The proportion of patients with EGC was 67.6% (95% confidence interval [CI], 58.0–76.0%) and 65.7% (95% CI, 61.3–69.9%) in the ≤24 months and >24 months groups, respectively. There was no significant difference in the proportion of patients with EGC between the two groups (*P* = 0.712). The power of the study for discriminating a difference in the proportion of EGC was 63.4%.

The proportion of lesions that fulfilled the expanded indications for ESD and lesions treated with ESD according to the interval between endoscopic examinations are shown in Fig 2B. There was no difference in the proportion of lesions that met the ESD indications according to the interval between endoscopic examinations (*P* = 0.430). However, the proportion of lesions that were treated with ESD did differ according to the interval between endoscopic examinations (≤24 months vs. >24 months; 9.8% vs. 4.5%, respectively; *P* = 0.032).

The mean lesion size was 23.8 mm (standard deviation [SD], 22.2 mm) in the ≤24 months group, while the mean lesion size in the >24 months group was 30.5 mm (SD, 23.1 mm). Lesion size was significantly smaller in the ≤24 months group than in the >24 month group (*P* = 0.008). Fig 2C also demonstrates the smaller lesion sizes in the ≤24 months group compared to the >24 months group.

In our multivariable analysis, the only factor associated with EGC was gastrointestinal symptoms at the time of diagnosis (*P* < 0.001; Table 2). The interval between endoscopic examinations did not influence the early diagnosis of gastric cancer (with >24 months as the reference group: ≤24 months, odds ratio [OR] = 0.939, 95% CI = 0.583–1.513). We also found that gastrointestinal symptoms and male sex were factors associated with the presence of lesions that fulfilled ESD indications (*P* = 0.001 and *P* = 0.008, respectively; Table 3). However, the interval between endoscopic examinations was not associated with the presence of lesions that met ESD indications. Although intervals of ≤24 months between endoscopic examinations tended to increase the proportion of EGCs that were treated with ESD, there was no significant difference (with >24 months as the reference group: ≤24 months, OR = 2.095, 95% CI = 0.935–4.692; Table 4).

**Table 2:** Factors associated with the diagnosis of early gastric cancers.

Variable	Adjusted OR	95% CI	*P*-value
Age, /yr	1.018	0.970–1.069	0.460
Male	1.246	0.866–1.792	0.236
First-degree relatives with gastric cancer	1.338	0.688–2.603	0.391
Gastrointestinal symptoms	0.366	0.246–0.544	< 0.001
Interval between endoscopic examinations			
≤ 24 months	0.939	0.583–1.513	0.796
> 24 months	1.000		

OR, odds ratio; CI, confidence interval.

**Table 3:** Factors associated with the possibility of endoscopic submucosal dissection.

Variable	Adjusted OR	95% CI	*P*-value
Age, /yr	1.009	0.957–1.065	0.73
Male	1.678	1.147–2.454	0.008
First-degree relatives with gastric cancer	1.033	0.531–2.008	0.925
Gastrointestinal symptoms	0.500	0.335–0.747	0.001
Interval between endoscopic examinations			
≤ 24 months	1.135	0.699–1.844	0.609
> 24 months	1.000		

OR, odds ratio; CI, confidence interval.

**Table 4:** Factors associated with gastric cancers treated with endoscopic submucosal dissection.

Variable	Adjusted OR	95% CI	*P*-value
Age, /yr	1.000	0.901–1.109	0.995
Male	1.572	0.745–3.315	0.235
First-degree relatives with gastric cancer	1.477	0.483–4.516	0.494
Gastrointestinal symptoms	0.488	0.218–1.092	0.052
Interval between endoscopic examinations			
≤ 24 months	2.095	0.935–4.692	0.072
> 24 months	1.000		

OR, odds ratio; CI, confidence interval.

## Discussion

In regions where *Helicobacter pylori* is endemic and gastric cancer is prevalent, gastric cancer screening programs are used to detect EGC [6,24]. In both Korea and Japan, screening for gastric cancer is currently recommended for individuals 40 years or older [6,24]. The incidence of gastric cancer is relatively low in people less than 40 years old [8,9], so screening this population may not be cost-effective [25]. Nevertheless, many young people undergo screening endoscopy in Korea [8,12]. Setting aside the issue of cost-effectiveness, we sought to ascertain whether periodic endoscopies helped detect gastric cancer at an early stage in a young population. One reason we asked this question was because gastric cancer shows different clinicopathologic features in young versus elderly patients [10,13–16].

In our study, we found that periodic endoscopies did not increase the proportion of patients with EGC in a young population. Therefore, we believe that there is a lack of evidence to support screening endoscopies for the early diagnosis of gastric cancer in a young population. However, this finding should be interpreted cautiously, because the sample size may have been insufficient to identify the possible benefits of periodic endoscopy. Although we recruited as many patients as possible, the number of young patients with gastric cancer is small even in Korea where gastric cancer is prevalent, and the retrospective power of the study was only 63.4%. Additionally, we demonstrated that periodic endoscopies did not facilitate the detection of EGC that fulfilled the indications for ESD. Rapid progression and a larger proportion of patients with undifferentiated cancer are potential reasons why screening endoscopies are futile in terms of the early diagnosis of gastric cancer in a young population [10,19].

In our study, only 31 (5.4%) lesions were treated with ESD, although 155 (27.2%) lesions met the indications for ESD. This discrepancy was caused by the difference between the clinical and pathological assessments for lesions. The decision to treat with ESD was made based on the clinical assessment including tumor size in gross findings, while the ESD indication criteria were based on the pathological examination of the ESD specimen. Therefore, ESD could not be performed even in lesions that were within the ESD indication, if the tumor size had been over-estimated. Such a discrepancy is usually emphasized in undifferentiated cancers which are relatively prevalent in young patients, compared to differentiated cancers. Because the margins of undifferentiated EGC are usually poorly defined, it is difficult to determine the exact lesion size and assess whether the lesion meets ESD indications by conventional white light endoscopy [26]. Even for lesions that fulfill ESD indications, the complete resection rate is lower for undifferentiated cancer than for differentiated cancer [27]. Therefore, only a select number of undifferentiated EGC lesions that were small in size were treated with ESD.

Our results suggest that periodic endoscopies might be beneficial for increasing the proportion of lesions treated with ESD. This result may be related to the increased detection of smaller gastric cancers at a smaller size in patients undergoing periodic endoscopy. Although periodic endoscopy did not increase the proportion of patients with lesions diagnosed as EGC, it did enable the detection of gastric cancer at a smaller size, which could facilitate treatment with ESD.

Previous studies have shown that the clinicopathologic features of gastric cancer differ between young and elderly patients. The young patient group included a higher proportion of female patients than elderly patient population [10,15]. In addition, younger patients often developed gastric cancer in the body of the stomach rather than in the antrum [16]. Our study also demonstrated that an increased proportion of gastric cancer in a young population occurred in females and involved tumors located in the middle third of the stomach. The proportion of gastric cancer patients who were 40 years or older who had first-degree relatives with gastric cancer has been reported to be 16 to 18% in Korea [7]; therefore, the fact that only 8.7% of the patients in our study had a family history of gastric cancer seemed relatively low. An additional difference between young and elderly populations may be the fact that periodic endoscopies were not shown to beneficial for the early detection of gastric cancer in young patients [7]. Gastric cancer in a young population may exhibit different pathophysiology, and more studies will be required to clarify these features.

Although this was the first study to evaluate the benefits of periodic endoscopies for gastric cancer screening in a young population, it had several limitations. We tried to contact as many patients as possible to minimize selection bias; however, several patients were excluded because of contact interruption. Most of the excluded patients were already dead at the time of our survey. Moreover, we only included patients who underwent curative treatment, since most patients with unresectable gastric cancer died before the start of this study. Therefore, caution is required in the interpretation of the results of the study. Although we showed that periodic endoscopy did not aid in the detection of resectable gastric cancer at an earlier stage in young patients, it is still doubtful whether periodic endoscopy can influence the incidence of unresectable gastric cancer in a young population. Additionally, since we used questionnaires rather than prospectively collected data, the possibility of recall bias cannot be excluded. Patients who have a low quality of life, or a higher cancer stage may be more likely to recall their symptoms at the time of diagnosis. However, patients who had no symptoms underwent diagnostic endoscopy in a health promotion center rather than in a hospital, while those with any gastrointestinal symptoms usually underwent endoscopy in a hospital for the purpose of evaluation for the symptoms. Patients could recall their symptom history because they usually remembered the institution (health promotion center vs. hospital) where they had been diagnosed with gastric cancer. Therefore we think that the recall bias may be low in this study. In addition, we could not check the quality of endoscopy because the penultimate endoscopy was performed in a health promotion center or other hospital rather than our hospital. Missed lesions in the penultimate endoscopy may affect the results of our study. However, a previous study on interval gastric cancer in Korea showed missed lesion of endoscopy was not common [28]. Finally, we could not evaluate the effect of periodic endoscopy on the survival of patients because endoscopy intervals were determined using a questionnaire survey of surviving patients. To remedy these limitations, our institute has initiated a prospective study of the timing of endoscopies in newly diagnosed gastric cancer patients. We hope that study will validate the findings of the present study.

Despite these limitations, we believe our data forms a basis for determining the benefits of periodic endoscopy aided in the detection of gastric cancer in a young population. Although periodic endoscopies helped in the detection of gastric cancer when lesions were smaller in size, they seemed not to increase the proportion of patients with EGC in young patients diagnosed with resectable gastric cancer. Screening endoscopy in a young population might have a minor role in the early diagnosis of gastric cancer even in regions where gastric cancer is prevalent.
